# The Distribution of Eight Antimicrobial Resistance Genes in *Streptococcus oralis*, *Streptococcus sanguinis*, and *Streptococcus gordonii* Strains Isolated from Dental Plaque as Oral Commensals

**DOI:** 10.3390/tropicalmed8110499

**Published:** 2023-11-16

**Authors:** Verónica Morales-Dorantes, Rubén Abraham Domínguez-Pérez, Rosa Martha Pérez-Serrano, Juan Carlos Solís-Sainz, Pablo García-Solís, León Francisco Espinosa-Cristóbal, Claudia Verónica Cabeza-Cabrera, José Luis Ayala-Herrera

**Affiliations:** 1 Laboratory of Multidisciplinary Dentistry Research, Faculty of Medicine, Universidad Autónoma de Querétaro, Santiago de Querétaro 76176, Mexico; 2 Laboratorio de Genética y Biología Molecular, Faculty of Medicine, Universidad Autónoma de Querétaro, Santiago de Querétaro 76176, Mexico; 3 Departamento de Investigación Biomédica, Faculty of Medicine, Universidad Autónoma de Querétaro, Santiago de Querétaro 76176, Mexico; 4 Programa de Maestría en Ciencias Odontológicas, Departamento de Estomatología, Instituto de Ciencias Biomédicas, Universidad Autónoma de Ciudad Juárez, Ciudad Juárez 32310, Mexico; 5 Clínica de la Licenciatura y Posgrados de Odontología, Faculty of Medicine, Universidad Autónoma de Querétaro, Santiago de Querétaro 76176, Mexico; 6 Dental School, Universidad La Salle Bajío, León 37150, Mexico

**Keywords:** antibiotic resistance, dental plaque, *Streptococcus mitis*, *Streptococcus gordonii*, *Streptococcus oralis*, *Streptococcus sanguinis*

## Abstract

It has been proposed that oral commensal bacteria are potential reservoirs of a wide variety of antimicrobial resistance genes (ARGs) and could be the source of pathogenic bacteria; however, there is scarce information regarding this. In this study, three common streptococci of the mitis group (*S. oralis*, *S. sanguinis*, and *S. gordonii*) isolated from dental plaque (DP) were screened to identify if they were frequent reservoirs of specific ARGs (*blaTEM*, *cfxA*, *tetM*, *tetW*, *tetQ*, *ermA*, *ermB*, and *ermC*). DP samples were collected from 80 adults; one part of the sample was cultured, and from the other part DNA was obtained for first screening of the three streptococci species and the ARGs of interest. Selected samples were plated and colonies were selected for molecular identification. Thirty identified species were screened for the presence of the ARGs. From those selected, all of the *S. sanguinis* and *S. oralis* carried at least three, while only 30% of *S. gordonii* strains carried three or more. The most prevalent were *tetM* in 73%, and *blaTEM* and *tetW* both in 66.6%. On the other hand, *ermA* and *cfxA* were not present. Oral streptococci from the mitis group could be considered frequent reservoirs of specifically *tetM*, *blaTEM*, and *tetW*. In contrast, these three species appear not to be reservoirs of *ermA* and *cfxA*.

## 1. Introduction

The human oral cavity contains a densely populated microbial ecosystem [1,2]. It is the second-most colonized environment by bacteria in the human body [3], with an estimated total of 700 species, including harmless symbionts, commensals, and opportunistic pathogens [4]. This great microbial diversity partly results from the many different ecological niches in the oral cavity, which provide different environments to bacteria, but also to selective pressures like dietary modifications, diseases, and antimicrobial exposure [5,6].

Streptococci are the most abundant inhabitants of oral microbial communities, including dental plaque (DP) [7,8,9], especially *Streptococcus oralis*, *Streptococcus sanguinis*, and *Streptococcus gordonii*, which are members of the mitis group [10]. They are common oral commensals that constitute part of all human oral cavities. These streptococci possess the ability to attach to components of the salivary pellicle as well as to other oral bacteria through adhesin proteins that are expressed on the cell surface, so together with many other oral microorganisms form multispecies biofilms [11]. In these biofilms, several species are arranged in close proximity, which frequently leads to the establishment of interactions such as quorum-sensing systems, food chains, and the exchange of virulence and antimicrobial resistance genes (ARGs) [12]. ARGs encode proteins that provide bacteria with distinct mechanisms of antimicrobial resistance. These genes could be transferred by many mechanisms [13] applied between different bacteria species, yeast and even archaea in biofilms [14]. Conjugation, transduction, and transformation are the most recognized as parts of the horizontal gene transfer. Conjugation requires physical contact between cells via a conjugation pilus through which a conjugative element, usually a plasmid or a transposon is transferred. Transduction is a mechanism mediated by independently replicating bacterial viruses called bacteriophages; for this process, the host and donor cells can be physically separated. Transformation implies the uptake of exogenous DNA from the environment; therefore, this mechanism does not require a living donor cell, is the best-characterized genetic transfer process among streptococci, and has been found in most groups [15] and been proposed as being ubiquitous in them [16]. In this mechanism, the streptococci enter into a physiological state of genetic competence; in this state, they become capable of natural genetic transformation, facilitating the acquisition of foreign DNA from the external environment [16,17] which makes them particularly capable of acquiring abundant and diverse ARGs of which they would become reservoirs.

In another way, pathogenic bacteria, in addition to possessing intrinsic resistance, can also acquire a variety of ARGs through these mechanisms and become resistant to multiple antimicrobials [18,19]. Antimicrobial resistance in pathogenic bacteria represents one of the most significant challenges to modern medicine worldwide; it has become a menace that requires attention and intervention. The intensive use of antimicrobials in medicine and dentistry and their excessive use in non-medical settings, such as animal farming and agriculture, are the main reasons for the rapid increase in antimicrobial resistance [20,21]. This phenomenon has led to a global health crisis as antimicrobials have become less effective and refractory infections have begun to spread. This complex problem involves several bacterial species, resistance and transfer mechanisms, and reservoirs. DP is an important reservoir for bacteria carrying ARGs, including those encoding resistance to commonly used antimicrobials such as beta-lactams, tetracyclines, and macrolides, but also to other antimicrobials that are not used as frequently [22,23].

Historically, the antimicrobial resistance of specific pathogenic oral bacteria [24,25] or from pathologic niches (necrotic root canal or periodontal pocket) has been widely studied [26,27,28]. However, in the last decade, there has been increased attention on studying commensal bacteria from the oral cavity as potential silent reservoirs of a wide variety of ARGs [29,30]. These commensals are a significant concern because they could be the source of ARGs to pathogenic species [7,13,31]. To address the antimicrobial resistance problem, it is essential to know the location of antimicrobial resistance reservoirs and the ARGs they contain to better predict the emerging resistance among pathogens [32,33] and to implement bacterial control mechanisms for pathogenic and commensal bacteria. In order to provide information regarding this, the objective of this investigation was to identify if three of the most common oral commensal streptococci (*S. oralis*, *S. sanguinis*, and *S. gordonii*), specifically of the mitis group, are frequent reservoirs of eight specific ARGs (*bla*TEM, *cfxA*, *tetM*, *tetW*, *tetQ*, *ermA*, ermB, and ermC) when they are present in the DP of adults with moderate caries.

## 2. Materials and Methods

### 2.1. Study Population and Clinical Evaluation

This cross-sectional study included 80 patients of both sexes between 18 and 65 years of age who met the selection criteria and were undergoing preventive dental treatment at the dental clinic in the Faculty of Medicine of the Autonomous University of Querétaro, México. Informed and voluntary written consent was obtained before the patient completed an oral and systemic health questionnaire and underwent a clinical examination. The study protocol was in accordance with the Declaration of Helsinki (2013 version) and was approved by the Ethical Committee of the Faculty of Medicine and the Research and Postgraduate Studies Council (FME-2022-01).

All patients were examined sitting on an ordinary dental chair, under standard dental light, using a plain mouth mirror, a North Carolina probe, and using air-drying by a single operator. The total visible surfaces of all teeth were assessed for dental caries. Only patients presenting at least 28 teeth and from 8 to 21 surfaces (for anterior teeth: mesial, buccal, distal, and palatine/lingual; for posterior teeth: mesial, buccal, distal, occlusal, and palatine/lingual) with caries were included to be classified as presenting moderate caries according to the Integrative Dental Caries Index (IDCI) [34]. Those who had undergone dental treatments, including prophylaxis, had received antimicrobial therapy in the previous 6 months or had brushed their teeth 3 h before sample collection were excluded. In addition, those who smoked, had periodontitis (determined when the pocket depth was >3 mm, and the attachment loss was ≥2 mm in at least 30% of the measured sites [35]), generalized gingivitis, symptomatic irreversible pulpitis, carious pulp exposure, abscesses or fistulae, and infectious diseases or chronic systemic diseases; and pregnant or lactating women were also excluded.

### 2.2. Sample Collection

A DP sample from the supragingival area was collected from 10 teeth of each patient (anterior, posterior, upper, and lower teeth) using a Gracey curette (Hu-Friedy Mfg. Co. LLC, Chicago, IL, USA). Each DP sample was deposited in a tube containing 6 mL of sterile brain heart infusion broth (BHI) and incubated for 24 h at 36 °C. Twenty-four hours later, the tube was centrifuged at 12,000× *g* for 5 min, and 5 mL of the supernatant was removed. The pellet was resuspended in the remaining 1 mL infusion. Five hundred microliters was deposited in a second tube with 6 mL of sterile BHI and stored at 36 °C until the possible selection to agar plate culture. The remaining 500 μL was deposited in a microcentrifuge tube containing 500 μL of sterile phosphate-buffered saline (PBS) solution and stored at −20 °C until deoxyribonucleic acid (DNA) purification.

### 2.3. Bacteria and ARG Identification in the DP Samples

DNA was obtained from each stored microcentrifuge tube as follows; the microtubes with the samples were centrifuged (16,000× *g* for 10 min) to obtain the cell pellet. After removing the supernatant, the pellet was washed three times with 1 mL of PBS (pH 7.4), resuspended in 200 μL of cell lysis buffer (1.0% Triton X- 100,20 mM Tris–HCl, 2 mM EDTA, pH 8.0) and incubated at 85 °C for 15 min. Then, 100 μL of 200 U/mL mutanolysin (Sigma, St. Louis, MO, USA) was added and incubated at 50 °C for 1 h, followed by treatment with 100 μL of protein precipitation solution (Puregene DNA isolation kit, Gentra Systems, Minneapolis, MN, USA). The proteins were removed by centrifugation (16,000× *g* for 10 min). The DNA was purified by phenol–chloroform–isoamyl alcohol (25:24:1, *v*/*v*; (Sigma, St. Louis, MO, USA) extraction and isopropanol (Sigma, St. Louis, MO, USA) precipitation. The extracted DNA was dissolved in 50 μL of sterile molecular biology-grade water. 

Three primer pairs were used to identify streptococci by using species-specific regions from the glucosyltransferase gene [36] while eight primer pairs were used to identify the ARGs [37]. Polymerase chain reaction (PCR) was performed in a total volume of 25 μL of a reaction mixture containing dNTPs, MgCl2, specific primers, DNA template, and Taq polymerase (Radiant, Alkali Scientific, Pompano Beach, FL, USA) in a 2720 thermal cycler (Applied Biosystems, Foster City, CA, USA) for 25 cycles: 30 s at 94 °C, 30 s at 50–70 °C (Table 1) and 30 s at 72 °C. PCR products were submitted to 2% agarose gel electrophoresis using Tris-boric acid-EDTA buffer and 1Kb Plus DNA marker (Invitrogen, Carlsbad, CA, USA). Each gel was stained with 0.5 μg/mL of ethidium bromide (Invitrogen, Carlsbad, CA, USA) and examined under ultraviolet light to determine whether the PCR produced bands of the expected sizes.

### 2.4. Culture and Identification of Streptococci from Selected DP Samples

The previously stored DP samples that simultaneously presented at least two species and at least four ARGs were selected for culturing. Each tube was vortexed, and an aliquot was inoculated onto trypticase soy with sucrose and bacitracin (TYS20B) agar plates. The plates were incubated in anaerobic conditions at 36 °C for 24–48 h. Once bacteria grew on the agar plates, 1 to 3 colonies with different phenotypes were randomly selected from each one and collected in microtubes containing sterile PBS for subsequent identification of streptococci. DNA was obtained from each one and PCR was run according to the previously reported methods and parameters. In addition, PCR products were submitted to electrophoresis as explained before.

### 2.5. Identification of ARGs in the Selected Isolated Streptococci

**Figure 1:**
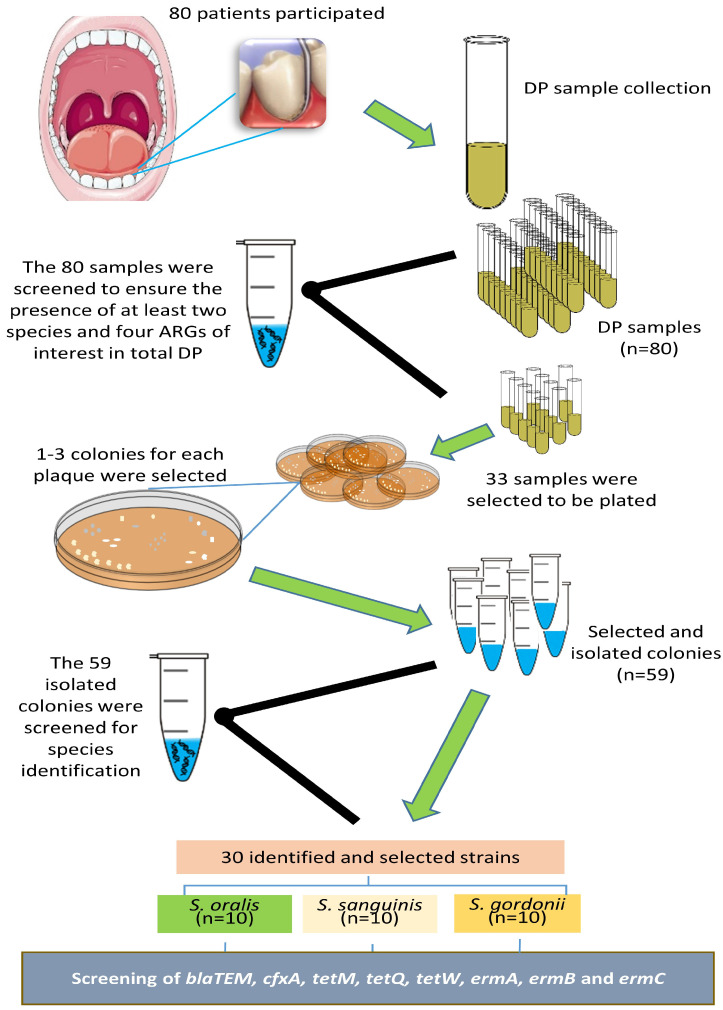
Flowchart of the complete process.

DNA of ten strains of each of the three identified species (*n* = 30) originating from DP of different patients were selected. In each of them, the eight ARGs were screened using the aforementioned primers, PCR and electrophoresis conditions. Figure 1 details a flowchart of the complete process.

### 2.6. Statistical Analysis

All data are presented in tables as a frequency and percentage. Differences in the distribution of the ARGs in the three streptococci were analyzed using the chi-square test with Graph-Pad Instat, version 3.0 (GraphPad Software, San Diego, CA, USA). Statistical significance was set at *p* < 0.05.

## 3. Results

The mean age of the 80 patients was 36.66 ± 11.01 (range 18–62) years; they were predominantly female (68.7%). Seventy-seven patients (96.2%) presented at least one species of interest in their DP (Table 2). Overall, 78.7% presented *S. oralis*, 70% *S. sanguinis*, and 68.7% *S. gordonii*.

Worryingly, 32.5% of the patients carried five or more ARGs in their DP, and only 11.2% were free of the eight ARGs (Table 3). *tetM* was the most frequent (86.2%), while *ermA* was the least frequent (10%) (Table 4).

Thirty-three DP samples simultaneously presenting at least two species of interest and at least four ARGs were chosen to be plated (six samples with six ARGs, twenty samples with five ARGs, and seven samples with four ARGs). From the 33 plates, 59 colonies were isolated and their DNA was submitted to PCR for species identification (Table 5). Of these strains, only the 10 identified as *S. oralis*, 10 as *S. sanguinis*, and 10 as *S. gordonii* were selected to be screened. 

In the 30 selected strains, the most prevalent ARGs were *tetM* in 73.3% and *blaTEM* and *tetW* in 66.6%. On the other hand, *ermA* and *cfxA* were not present. Eighty percent of *S. sanguinis* strains and 70% of *S. oralis* strains carried three or more ARGs, while only 30% of *S. gordonii* strains carried three or more ARGs (Table 6). However, there was no significant difference in the distribution of the ARGs in the three species (*p* = 0.8124) nor in the distribution (*p* = 0.0603) of the number of simultaneously detected genes (Table 7).

## 4. Discussion

It has been proven that oral bacteria carry several ARGs [13,22] and commensal bacteria may act as silent reservoirs of them [29,30]. Streptococci are mostly recognized as commensals in the oral cavity, especially those of the mitis group. Furthermore, they are the most abundant species in the oral microbiota, so the presence of ARGs in them represents an important source of pathogenic bacteria. Hence, it is important to identify which ARGs are carried by some specific representative streptococci in the supragingival DP samples of a common population. To achieve this, we first screened a group of patients who were diagnosed with moderate caries according to the IDCI but were free of other oral and systemic diseases. We established several selection criteria to control a large number of variables and to produce a homogeneous population. This approach also allowed us to avoid as much as possible the presence of pathogenic communities in the oral cavity or the consumption of antimicrobials.

During the process, to obtain the 30 different strains of the three species of interest, additional information was obtained. Mainly, the prevalence of *S. oralis*, *S. sanguinis*, *S. gordonii*, and the eight ARGs in the DP of the 80 patients. Interestingly, of all the DP samples, only 45% simultaneously presented the three streptococci species, which are widely reported as present in DP [10], while only three patients did not present any of them. Otherwise, only nine patients (11.2%) tested negative for all the tested ARGs, while 26 patients (32.5%) carried five to six of the ARGs. This fact is striking because in a similar group of patients [26] taken from the same university clinic but in a well-established pathological niche (necrotic root canals with apical periodontitis), only 3.3% carried five to six of these same ARGs. This clearly shows that DP should be considered as an extensive reservoir of ARGs, even more than the microbiota of a well-established pathological niche.

The *tetM* gene, a member of the *tet* family that encodes a ribosomal protection protein that provides tetracycline resistance, was the most prevalent ARG, present in 86.2% of the DP samples. This finding coincides with previous reports that recognized it as the most widespread *tet* gene in the oral microbiota [23], the most prevalent specifically in supragingival DP [38,39], and present in 94% of saliva samples [40]. This ARG is typically present on conjugative transposons of the Tn916/Tn1545 family [38], which usually also contains the *ermB* gene, from the *erm* family that confers resistance to macrolides via methylation of the ribosome [41,42]. In this group of patients, the *ermB* gene was the second most prevalent ARG (57.5%) and was present simultaneously with the *tetM* in 44 (55%) DP samples. *blaTEM*, an ARG related to beta-lactam resistance, was the third most prevalent ARG (48.7%), a finding that coincides (46.2%) with a previous study that examined DP samples from adult patients [43]. 

It is of clinical importance that the three most prevalent ARGs in the 80 patients are related to providing resistance to three commonly used groups of antimicrobials: beta-lactams, tetracyclines, and macrolides; this is undoubtedly related to the rapid and uncontrolled emergence of antimicrobial resistance as a severe public health problem [44].

When analyzing the results obtained from the 30 isolated and identified strains, we observed that *tetM*, *blaTEM*, and *tetW* were carried with a high frequency (>65%), which may indicate that *S. oralis*, *S. sanguinis*, and *S. gordonii* are frequent reservoirs of these ARGs. The *ermB* was carried infrequently (23.3%) and only six (20%) of the strains carried the *ermB* and *tetM* genes simultaneously, as previously reported [41,42]: one *S. oralis*, two *S. gordonii*, and three *S. sanguinis*. The *cfxA* gene, responsible for the resistance to penicillins and cephalosporins [45], and the *ermA* gene were never carried by these three species, which could partly explain why these ARGs were also the least prevalent in the 80 DP samples.

Regarding the diversity of ARGs carried by each of the screened species in this investigation, *S. sanguinis* and *S. oralis* carried three to four ARGs with high frequency (70%). In comparison, *S. gordonii* presented three to four ARGs in only 20%. *S. sanguinis* and *S. oralis* seem to be more robust reservoirs of the screened ARGs, while *S. gordonii* seems to be a weaker reservoir. Moreover, it is also striking that 100% of the *S. sanguinis* strains carried the *tetM* gene.

Our data confirm that these oral commensal streptococci carry a diverse array of ARGs. This is of particular clinical importance and is worrisome in several ways. First, there is sufficient evidence of inter- and intra-genus transfer of ARGs in the oral cavity. Indeed, the transfer of ARGs between different genera is bidirectional; for example, it has been shown between *S. gordonii* and *Enterococcus faecalis* in root canals [46]. Also, when present in the same niche, *S. mitis*, *S. oralis*, and *S. gordonii* can provide *Streptococcus pneumoniae* with “new genes” [47,48]. In addition, gene transfer between species has led to the proposal that the resistance profiles of some streptococci can be used as a markers for the risk of the emergence of resistance in a given bacterial population of, for example, *Streptococcus pyogenes* or *S. pneumoniae* [48,49]. 

Second, as if the carriage of a substantial diversity of ARGs by the streptococci as part of the oral resistome was not enough, it is important to remember that several of them, although considered oral commensals, can behave as opportunistic pathogens and can cause severe infections in other body sites [50]. The oral cavity provides a wide portal to the rest of the body through the respiratory or digestive tract as well as the gingival surface following cuts or abrasions sustained from brushing or eating, leading to bacteremia [51]. Some of them possess pathogenic abilities that could cause invasive infections such as infective endocarditis, septicemia and pneumonia [52,53,54], which would become even more complex if they presented antimicrobial resistance.

For this study, the most important limitation is the small number of screened strains—only 10 of each species; this small number limited our ability to draw conclusions. However, despite this limitation, the population from which they were isolated was well controlled, without underlying diseases or other confounders. Another important limitation is the technique used to screen the antimicrobial resistance. Despite the advantages of studying resistance genotypes, there are also disadvantages; the main one is that detecting ARGs in a strain only indicates a “potential resistance” since these genes may not be expressed or may have mutated to a nonfunctional form. These situations cause the genotype of resistance to not coincide with the resistance phenotype [55]. Fortunately, positive correlations have been reported between genotypic and phenotypic resistance, so the so-called “potential resistance” is highly coincident [56].

In addition, an important strength of this investigation is the use of eight pairs of primer sequences to identify common and widely reported ARGs, which not only allows for a panoramic view of the resistance profile of three critical groups of antimicrobials in each streptococci species, but also knowledge about the resistance profile present in the complete DP of the patients through the screening of the same eight ARGs in it, taking into consideration that screening ARGs in the complete microbiota of a niche provides more detailed and complete clinical information because of the inclusion of a large number of non-cultivable bacteria in these niches.

Furthermore, this study evidences the clinical importance of continually removing DP through brushing given that DP plays an essential role in antimicrobial resistance. DP maintained in the mouth provides a reservoir of ARGs that could promote their spread locally and systemically in the individual, but also in the community where the individual lives, since it has been reported that resistance profiles of oral bacteria can be shared between humans and even their pets [57]. Good oral hygiene is crucial not only to prevent oral or systemic diseases, but also because the simple practice of continually removing the DP has an impact against the spread of antimicrobial resistance.

## 5. Conclusions

Oral commensal streptococci from the mitis group (*S. oralis*, *S. sanguinis*, and *S. gordonii*) isolated from DP are frequent reservoirs of three of the eight screened ARGs (*tetM*, *blaTEM*, and *tetW*). In contrast, these three species appear not to be reservoirs of *ermA* and *cfxA*.

## Figures and Tables

**Table 1:** Primer sequences and conditions to identify the three streptococci species and the antimicrobial resistance genes.

Streptococci Species	Primer Sequence (5′-3′)	Annealing Temp (°C)	Amplicon Size (bp)
*S. sanguinis*	GGATAGTGGCTCAGGGCAGCCAGTT GAACAGTTGCTGGACTTGCTTCTC	70	313
*S. oralis*	TCCCGGTCAGCAAACTCCAGCC GCAACCTTTGGGATTTGCAAC	66	374
*S. gordonii*	CTATGCGGATGATGCTAATCAAGTG GGAGYCGCTATAATCTTGTCAGAAA	55	440
**ARGs**	**Primer Sequence (5′-3′)**	**Annealing** **Temp (°C)**	**Amplicon** **Size (bp)**
*blaTEM*	CCAATGCTTAATCAGTGAGG ATGAGTATTCAACATTTCCG	50	858
*cfxA*	GCGCAAATCCTCCTTTAACAA ACCGCCACACCAATTTCG	55	802
*tetM*	GTGGACAAAGGTAC AACGAG CGGTAAAGTTCGTCACACAC	55	406
*tetW*	GAGAGCCTGCTATATGCCAGC GGGCGTATCCACAATGTTAAC	55	168
*tetQ*	TTATACTTCCTCCGGC ATCG ATCGGTTCGAGAATGTCCAC	55	904
*ermA*	AACACCCTGAACCCAAGGGACG CTTCACATCCGGATTCGCTCGA	50	420
*ermB*	GAAAAGGTACTCAACCAAATA AGTAACGGTACTTAAATTGTTTAC	55	639
*ermC*	AATC GGCTCAGGAAAAGG ATCGTCAATTCCTGCATG	50	562

The primer sequences are described in [36,37].

**Table 2:** Frequency of simultaneously detected streptococci species in the DP (*n* = 80).

Number of Species	Frequency (%)
Three	36 (45)
Two	25 (31.2)
One	16 (20)
None	3 (3.7)

**Table 3:** Frequency of simultaneously detected antimicrobial resistance genes in DP samples (*n* = 80).

Number of ARGs	Frequency (%)
Eight	0
Seven	0
Six	6 (7.5)
Five	20 (25)
Four	13 (16.2)
Three	11 (13.7)
Two	12 (15)
One	9 (11.2)
None	9 (11.2)

**Table 4:** Frequency of detected antimicrobial resistance genes in DP samples (*n* = 80).

Specific ARG	Frequency (%)
*tetM*	69 (86.2)
*ermB*	46 (57.5)
*blaTEM*	39 (48.7)
*tetW*	33 (41.2)
*ermC*	24 (30)
*tetQ*	19 (23.7)
*cfxA*	11 (13.7)
*ermA*	8 (10)

**Table 5:** Distribution of the 59 cultured and identified species.

*S. oralis*	10
*S. sanguinis*	11
*S. gordonii*	16
Others	22

**Table 6:** Frequency of antimicrobial resistance genes in each of the species.

	*S. sanguinis* (*n* = 10)	*S. oralis* (*n* = 10)	*S. gordonii* (*n* = 10)	All Selected (*n* = 30)
Specific ARG	Frequency (%)			
*tetM*	10 (100)	6 (60)	6 (60)	22 (73.3)
*blaTEM*	8 (80)	8 (80)	4 (40)	20 (66.6)
*tetW*	6 (60)	8 (80)	6 (60)	20 (66.6)
*ermC*	3 (30)	6 (60)	2 (20)	11 (36.6)
*ermB*	3 (30)	2 (20)	2 (20)	7 (23.3)
*tetQ*	2 (20)	0	2 (20)	4 (13.3)
*ermA*	0	0	0	0
*cfxA*	0	0	0	0

Distribution comparison of the ARGs in the three streptococci species (*p* = 0.8124; Chi-square).

**Table 7:** Frequency of simultaneously detected antimicrobial resistance genes in each species.

Number of Detected ARGs	Frequency (%)			
Six	1 (10)	0	1 (10)	2 (6.6)
Five	0	0	0	0
Four	1 (10)	6 (60)	1 (10)	8 (24.2)
Three	6 (60)	1 (10)	1 (10)	8 (24.2)
Two	2 (20)	1 (10)	2 (20)	5 (16.6)
One	0	1 (10)	2 (20)	3 (10)
None	0	1 (10)	2 (20)	3 (10)

Distribution comparison by number of simultaneously detected genes (*p* = 0.0603; Chi-square).

## Data Availability

The dataset used and analyzed during this study is available from the corresponding author on reasonable request.
